# A Case Report on Fibromuscular Dysplasia With Extrarenal Involvement

**DOI:** 10.7759/cureus.50358

**Published:** 2023-12-11

**Authors:** Hussain A Al Mubarak, Shalan M Alshalan, Natheer B Alkhunaizi, Abdullah H Alorabi, Ahmad M Abdulfatah

**Affiliations:** 1 General Practice, Batterjee Medical College, Jeddah, SAU; 2 General Practice, Ibn Sina National College for Medical Studies, Jeddah, SAU; 3 Surgery, Dallah Hospital, Riyadh, SAU

**Keywords:** computed tomography angiography, mesenteric artery stenosis, renal artery aneurysm, acute abdominal pain, fibromuscular dysplasia

## Abstract

Fibromuscular dysplasia is a vascular disorder characterized by nonatherosclerotic, noninflammatory arterial abnormalities, primarily affecting renal arteries. While extrarenal involvement is rare, it poses diagnostic challenges due to diverse clinical presentations. We present the case of a middle-aged female who presented with sudden-onset severe abdominal pain and hypertension. Physical examination revealed tenderness, guarding, and hypertensive urgency. Diagnostic workup, including laboratory investigations and computed tomography, identified multifocal arterial stenosis in both renal and mesenteric arteries, consistent with fibromuscular dysplasia. A multidisciplinary team initiated antihypertensive medications and performed angioplasty to address vascular stenosis. The patient showed significant improvement post intervention, highlighting the efficacy of a comprehensive management strategy. This case underscores the clinical complexities of fibromuscular dysplasia, emphasizing the diagnostic challenges posed by extrarenal manifestations. The successful multidisciplinary intervention highlights the importance of timely and targeted measures in addressing both renal and extrarenal complications.

## Introduction

Fibromuscular dysplasia is a nonatherosclerotic, noninflammatory vascular disorder characterized by abnormal cellular proliferation within the arterial walls, leading to stenosis and aneurysmal dilation [1]. While fibromuscular dysplasia predominantly affects renal arteries, the involvement of extrarenal arteries, including those supplying the gastrointestinal tract, is a rare manifestation. The pathophysiology of fibromuscular dysplasia remains elusive, with genetic, hormonal, and mechanical factors implicated in its etiology [1]. Clinically, fibromuscular dysplasia often presents as renovascular hypertension, and its extrarenal involvement can manifest as abdominal pain due to mesenteric arterial stenosis. Additionally, fibromuscular dysplasia may affect various other arterial sites, contributing to a diverse clinical spectrum. The extrarenal sites commonly implicated include but are not limited to the carotid, vertebral, and iliac arteries [1,2]. The rarity and diverse clinical presentations of extrarenal fibromuscular dysplasia pose diagnostic challenges, necessitating a high index of suspicion for accurate and timely identification [2].

The current literature on fibromuscular dysplasia primarily focuses on renal artery involvement, with limited emphasis on extrarenal manifestations [2]. Understanding the broader spectrum of fibromuscular dysplasia, particularly its impact on intestinal arteries, is crucial for comprehensive patient management. This case highlights the importance of considering fibromuscular dysplasia in the differential diagnosis of acute abdominal pain associated with hypertension. Other potential differentials in such presentations include renal artery stenosis, pheochromocytoma, abdominal aortic aneurysm, and coarctation of the aorta [1,2]. A thorough evaluation is crucial to distinguish between these conditions and facilitate prompt and accurate diagnosis and management.

## Case presentation

A 45-year-old female, previously in good health, presented to the emergency department with a sudden onset of severe abdominal pain. The patient reported the pain as crampy and diffuse, primarily localized in the flank regions, and radiating to the lower abdomen. She denied any associated symptoms such as nausea, vomiting, changes in bowel habits, or urinary symptoms. There was no recent history of trauma, fever, or other systemic symptoms.

Upon further inquiry into her medical history, the patient reported no significant past illnesses, surgeries, or chronic medications. She had no known cardiovascular risk factors such as diabetes or hyperlipidemia. The patient had a regular menstrual cycle, and there were no reported gynecological issues.

Physical examination revealed a middle-aged female in acute distress, with tenderness and guarding over the entire abdomen. Vital signs were notable for hypertension, with a blood pressure reading of 160/90 mmHg. The heart rate was within normal limits, and there were no signs of dehydration or shock. Bowel sounds were present, and no abdominal masses were palpable. The rest of the systemic examination was unremarkable.

Given the severity of the abdominal pain and the presence of hypertension, a comprehensive workup was initiated. Laboratory investigations, including a complete blood count, urea and electrolytes, and serum amylase, revealed normal results (Table 1). Urinalysis ruled out any renal pathology. To comprehensively evaluate potential vasculitis as a cause, a vasculitis panel was conducted, encompassing relevant serological markers such as antineutrophil cytoplasmic antibodies, erythrocyte sedimentation rate, and C-reactive protein. The results of the vasculitis panel were within normal limits, further supporting the exclusion of vasculitis in this case.

**Table 1:** Summary of initial laboratory findings

Laboratory Parameter	Result	Reference Range
White Blood Cell Count	7,800/μL	4,000-11,000/μL
Hemoglobin	13.5 g/dL	12-16 g/dL
Platelet Count	250,000/μL	150,000-450,000/μL
Sodium	138 mEq/L	135-145 mEq/L
Potassium	4.2 mEq/L	3.5-5.0 mEq/L
Chloride	100 mEq/L	98-106 mEq/L
Bicarbonate	26 mEq/L	22-30 mEq/L
Serum Amylase	50 U/L	25-125 U/L

**Figure 1:**
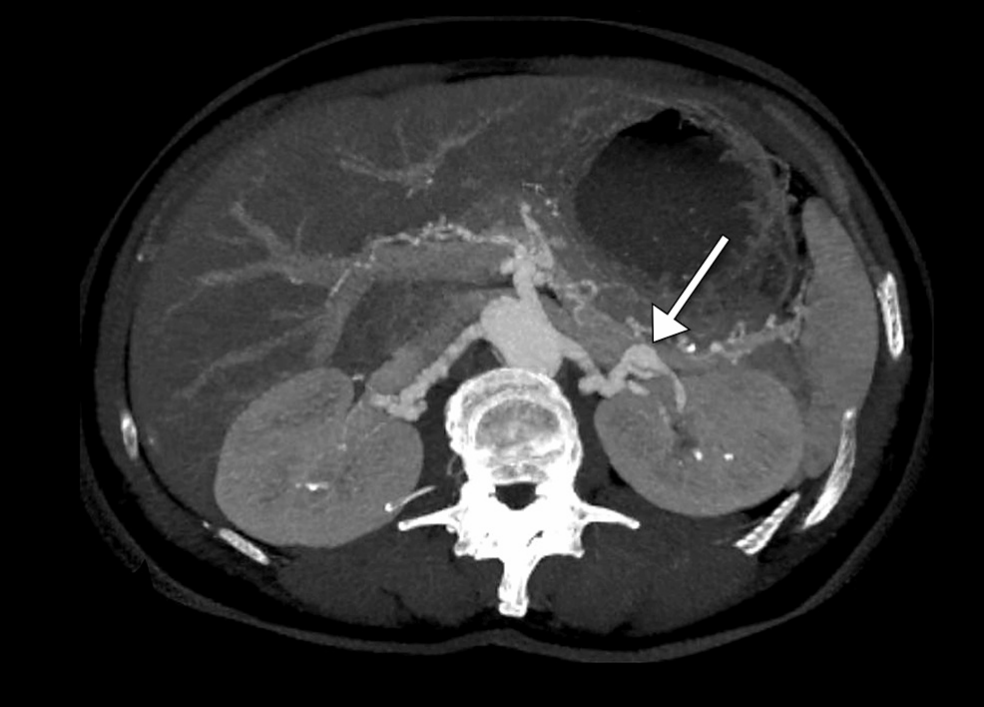
Axial CTA image of the abdomen showing left renal artery aneurysm (arrow) associated with a beaded appearance of renal and mesenteric arteries, consistent with fibromuscular dysplasia CTA: computed tomography angiography

The computed tomography scan revealed an evidence of multifocal arterial stenosis affecting the renal arteries bilaterally, consistent with fibromuscular dysplasia. Additionally, the mesenteric arteries displayed similar findings. No other abnormalities were noted (Figure 1).

A multidisciplinary team, including vascular surgeons, nephrologists, and gastroenterologists, collaborated to outline a comprehensive management plan. The patient was initiated on antihypertensive medications to manage her blood pressure and optimize renal perfusion. Surgical intervention, including angioplasty of the affected arteries, was deemed necessary to alleviate the vascular stenosis.

The hospital course was marked by close monitoring of the patient's renal function, blood pressure control, and symptomatic relief of abdominal pain. Postoperatively, the patient showed significant improvement in her symptoms. As the patient stabilized, she was transitioned to outpatient care with ongoing follow-up. Long-term management included continued blood pressure control, antiplatelet therapy, and regular surveillance to monitor for any recurrence or complications related to fibromuscular dysplasia.

## Discussion

The presented case of fibromuscular dysplasia involving not only renal but also intestinal arteries underscores the complexity and heterogeneity of this vascular disorder. While fibromuscular dysplasia primarily affects renal arteries, our case highlights the importance of considering extrarenal manifestations, particularly in the context of acute abdominal pain and hypertension. The diverse clinical presentations of fibromuscular dysplasia pose diagnostic challenges, necessitating a multidisciplinary approach to ensure accurate identification and tailored management [1]. This case contributes to the limited body of literature on extrarenal fibromuscular dysplasia, emphasizing the need for heightened clinical awareness and thorough diagnostic evaluation in cases of acute abdominal pain, especially when accompanied by hypertensive urgency.

The pathophysiology of fibromuscular dysplasia remains incompletely understood, and the mechanisms driving its extrarenal involvement, such as in the mesenteric arteries, are particularly enigmatic [3]. Genetic predisposition, hormonal influences, and mechanical factors have been implicated in fibromuscular dysplasia's etiology, but the specific interplay leading to extrarenal manifestations remains an area of ongoing research. Future investigations should aim to unravel the molecular and cellular pathways contributing to fibromuscular dysplasia's diverse clinical presentations, shedding light on potential therapeutic targets for this intricate vascular disorder [3,4].

The management of fibromuscular dysplasia necessitates a comprehensive and individualized approach, as highlighted in this case [5]. In addition to blood pressure control and antihypertensive medications, the patient underwent surgical intervention with angioplasty to address the arterial stenosis [4,5]. The successful revascularization of both renal arteries resulted in significant symptom relief, emphasizing the efficacy of a multidisciplinary strategy in managing complex cases of fibromuscular dysplasia. This case adds to the evolving landscape of fibromuscular dysplasia management, showcasing the potential benefits of timely and targeted interventions to mitigate both renal and extrarenal complications.

## Conclusions

In conclusion, this case underscores the clinical challenges and therapeutic considerations associated with fibromuscular dysplasia involving both renal and intestinal arteries. The effective resolution of symptoms following a multidisciplinary intervention highlights the critical importance of timely and targeted measures in addressing the complexities of fibromuscular dysplasia. This case contributes to the evolving understanding of extrarenal fibromuscular dysplasia, emphasizing the need for heightened clinical awareness in cases of acute abdominal pain associated with hypertension.
